# Healing Kinetics of Sinus Lift Augmentation Using Biphasic Calcium Phosphate Granules: A Case Series in Humans

**DOI:** 10.3390/bioengineering12080848

**Published:** 2025-08-06

**Authors:** Michele Furlani, Valentina Notarstefano, Nicole Riberti, Emira D’Amico, Tania Vanessa Pierfelice, Carlo Mangano, Elisabetta Giorgini, Giovanna Iezzi, Alessandra Giuliani

**Affiliations:** 1 Biomedical Science and Public Health Department, Polytechnic University of Marche, 60126 Ancona, Italy; m.furlani@staff.univpm.it; 2 Life and Environmental Sciences Department, Polytechnic University of Marche, 60131 Ancona, Italy; vnotarstefano@unite.it (V.N.); e.giorgini@staff.univpm.it (E.G.); 3 Department of Bioscience and Technology for Agriculture, Food and Environment, University of Teramo, 64100 Teramo, Italy; 4 Department of Clinical and Molecular Sciences, Polytechnic University of Marche, 60126 Ancona, Italy; n.riberti@staff.univpm.it; 5 Medical, Oral and Biotechnological Sciences Department, University “G. D’Annunzio” of Chieti-Pescara, 66013 Chieti, Italy; emira.damico@unich.it (E.D.); tania.pierfelice@unich.it (T.V.P.); gio.iezzi@unich.it (G.I.); 6 Department of Dental Sciences, San Raffaele University, 20132 Milan, Italy; camangan@gmail.com; 7 Odontostomatologic and Specialized Clinical Sciences Department, Polytechnic University of Marche, 60131 Ancona, Italy

**Keywords:** bone regeneration, biphasic calcium phosphate, sinus lift augmentation, micro computed tomography, Raman microspectroscopy, bone healing

## Abstract

Sinus augmentation provides a well-established model for investigating the three-dimensional morphometry and macromolecular dynamics of bone regeneration, particularly when using biphasic calcium phosphate (BCP) graft substitutes. This case series included six biopsies from patients who underwent maxillary sinus augmentation using BCP granules composed of 30% hydroxyapatite (HA) and 70% β-tricalcium phosphate (β-TCP). Bone core biopsies were obtained at healing times of 6 months, 9 months, and 12 months. Histological evaluation yielded qualitative and quantitative insights into new bone distribution, while micro-computed tomography (micro-CT) and Raman microspectroscopy (RMS) were employed to assess the three-dimensional architecture and macromolecular composition of the regenerated bone. Micro-CT analysis revealed progressive maturation of the regenerated bone microstructure over time. At 6 months, the apical regenerated area exhibited a significantly higher mineralized volume fraction (58 ± 5%) compared to the basal native bone (44 ± 11%; *p* = 0.0170), as well as significantly reduced trabecular spacing (Tb.Sp: 187 ± 70 µm vs. 325 ± 96 µm; *p* = 0.0155) and degree of anisotropy (DA: 0.37 ± 0.05 vs. 0.73 ± 0.03; *p* < 0.0001). By 12 months, the mineralized volume fraction in the regenerated area (53 ± 5%) was statistically comparable to basal bone (44 ± 3%; *p* > 0.05), while Tb.Sp (211 ± 20 µm) and DA (0.23 ± 0.09) remained significantly lower (Tb.Sp: 395 ± 41 µm, *p* = 0.0041; DA: 0.46 ± 0.04, *p* = 0.0001), indicating continued structural remodelling and organization. Raman microspectroscopy further revealed dynamic macromolecular changes during healing. Characteristic β-TCP peaks (e.g., 1315, 1380, 1483 cm^−1^) progressively diminished over time and were completely absent in the regenerated tissue at 12 months, contrasting with their partial presence at 6 months. Simultaneously, increased intensity of collagen-specific bands (e.g., Amide I at 1661 cm^−1^, Amide III at 1250 cm^−1^) and carbonate peaks (1065 cm^−1^) reflected active matrix formation and mineralization. Overall, this case series provides qualitative and quantitative evidence that bone regeneration and integration of BCP granules in sinus augmentation continues beyond 6 months, with ongoing maturation observed up to 12 months post-grafting.

## 1. Introduction

Restoring dentition in edentulous patients with dental implants frequently presents substantial challenges, primarily due to insufficient alveolar bone volume [1]. This common deficiency often results from alveolar atrophy and the pneumatization of the maxillary sinus [2]. In such anatomically compromised sites, achieving adequate primary stability for dental implants is often hindered by a lack of sufficient basal bone density [3]. To overcome these limitations, biomaterials play a crucial role in augmenting the body’s intrinsic capacity for bone regeneration, providing the necessary scaffold to facilitate and sustain the regenerative cascade of newly forming bone.

Extensive research has explored a diverse array of biomaterials for bone regeneration procedures. These include, but are not limited to, demineralized freeze-dried bone allografts, calcium carbonate, bioactive glass, various polymers such as polylactic acid (PLA) and polyglycolic acid (PGA), bovine-derived xenografts and peptides [4,5,6], calcium sulfate, bovine deproteinized bone, and hydroxyapatite. While autologous bone remains the gold standard owing to its superior osteogenic potential, its clinical application is constrained by significant drawbacks, including donor site morbidity, limited availability, and the requirement for additional surgical interventions [7]. Consequently, the development of synthetic biomaterials has aimed to surmount these limitations [8]. Furthermore, concerns regarding the potential for disease transmission associated with allografts and xenografts have propelled research into synthetic bone substitutes specifically designed to mimic the physical and chemical attributes of native bone tissue, with the overarching objectives of achieving osteoconduction, osteoinduction, and osseointegration [9,10].

An ideal biomaterial for bone regeneration must exhibit a specific spectrum of biological and clinical characteristics. Biologically, it should actively recruit mesenchymal cells via host-derived growth factors and exert bioactive effects to promote ossification [11]. Moreover, it must furnish a three-dimensional scaffold that supports vascular ingrowth and the migration of osteoprogenitor cells, while being progressively bioresorbable over time. From a clinical standpoint, the material should be user-friendly, cost-effective, and radiographically evaluable throughout the healing process: indeed, to facilitate effective monitoring of its resorption and subsequent replacement by host bone, the biomaterial should possess radiopacity.

The intricate process of bone regeneration within a scaffold encompasses cellular recruitment, infiltration from surrounding bone tissue, and vascularization [12,13]. Numerous studies have consistently demonstrated that high porosity significantly enhances osteogenesis. Bioceramics, which structurally emulate natural bone, effectively integrate favorable mechanical properties with an interconnected porous architecture, making them suitable for delivering nutrients to cells. Structurally, pores larger than 100–150 µm are imperative to ensure adequate vascularization and tissue ingrowth [14]. Furthermore, an optimal biomaterial should degrade gradually, ideally being entirely supplanted by vital bone tissue, with its resorption rate precisely synchronized with the rate of new bone formation; indeed, excessively rapid degradation can substantially compromise regenerative outcomes [15,16].

However, in highly demanding defect morphologies, the inherent degradation rate of some biomaterials may be too high to ensure sufficient mechanical support at the augmented site [17]. In these specific cases, a substitute material with minimal or no degradation is often preferred to preserve the augmented volume [18]. Calcium phosphate (CP) in the form of hydroxyapatite (HA), possessing a Ca/P ratio of 1.67, precisely matches the inorganic phase of bone’s crystallographic structure. Generally, HA-based bone substitute materials are considered non-resorbable [19]. In contrast, tricalcium phosphate (TCP), with a Ca/P ratio of 1.5, more closely approximates the composition of the amorphous biological precursor to bone [20]. In bone defects exhibiting high osteogenic potential, TCP is known to be rapidly replaced by newly formed bone [21,22]. By combining HA and TCP to form biphasic calcium phosphate (BCP) and by carefully modulating the HA/TCP ratio, it has become possible to fine-tune both the degradation rate and the bioactivity of these materials [23,24,25].

Several studies have specifically investigated the bone regenerative potential of biphasic calcium phosphate ceramics in the HA/β-TCP ratio equal to 30/70. These investigations have assessed histological and histomorphometric results from human specimens retrieved from sinuses augmented with HA/β-TCP 30/70, comparing them to anorganic bovine bone (ABB), mineralized solvent-dehydrated bone allograft (MSDBA), and equine bone (EB) after various healing periods. Collectively, these observations strongly support the suitability of HA-TCP (30/70) for successful maxillary sinus augmentation procedures, positioning it as a highly promising option [26,27].

This multidisciplinary experimental study endeavors to shed light on the microarchitectural and macromolecular kinetics of biphasic (30% HA/70% TCP) β-tricalcium phosphate granules, specifically designed for novel strategies in jawbone engineering. The performance of these granules is investigated here within a clinical case series of sinus lift augmentation.

## 2. Materials and Methods

### 2.1. Sampling

The archives of the Implant Recovery Center of the Department of Medical, Oral, and Biotechnological Sciences at the University of Chieti-Pescara, Italy, were examined to identify biopsies obtained from routine maxillary sinus augmentation procedures in atrophic posterior maxillae utilizing synthetic micro-macroporous biphasic calcium phosphate (BCP) granules, composed of 70% beta-tricalcium phosphate and 30% hydroxyapatite (BTK, Dueville, Vicenza, Italy). The sinus augmentation procedures adhered to the classical lateral window technique as described by Tatum OH (1986) [28]. Only biopsies collected by a single surgeon (C.M.) were included.

### 2.2. Patient Selection

All patients requiring the extraction of a hopeless tooth, followed by a sinus augmentation procedure and subsequent implant-prosthetic rehabilitation, were considered potentially eligible for inclusion in the study.

The inclusion criteria were as follows: (i) age greater than 18 years; (ii) indication for the extraction of a hopeless tooth, subsequent sinus augmentation, and implant-prosthetic rehabilitation; and (iii) willingness to participate in the study.

Patients with uncontrolled systemic pathologies or periodontal disease, those undergoing or having undergone treatments with medications or radiotherapy that could potentially impair the healing of hard and soft tissues, as well as women who were pregnant or breastfeeding, were excluded from eligibility.

A total of six clinical cases were analyzed: three cases with biopsies harvested after a healing period of 9 months, two cases after 9 months, and one case after 12 months. Demographic data of the study and patient medical records are reported in Table 1. It is worth noting that cases 2 and 5 derive from the same patient, who underwent bilateral biopsies.

### 2.3. Ethical Approval

The study was conducted in full accordance with the ethical principles of the Declaration of Helsinki (2013 version) and received independent approval by the Territorial Ethics Committee of the Abruzzo Region (C.E.t.R.A.) (3rd amendment approval n. 22, 9 May 2024 of BONEISTO study). All patients received comprehensive information regarding the study protocol and provided written informed consent to participate.

### 2.4. Sample Processing and Analysis

**Figure 1:**
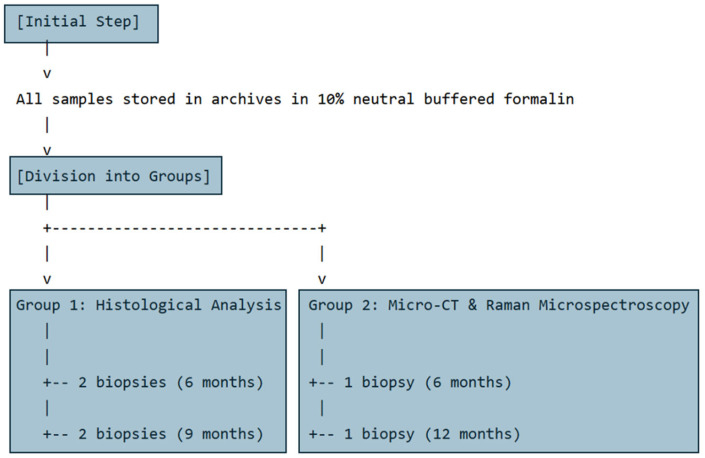
Chart summarizing case groups, analysis types, and healing time points.

All samples were stored in the archives in 10% neutral buffered formalin until experimental analysis. Subsequently, the samples were divided into two groups: Group 1, comprising cases 1–4 (cases 1, 2: 6 months of healing; cases 3, 4: 9 months of healing), designated for histological analysis; and Group 2, comprising cases 5 and 6 (case 5: 6 months of healing; case 6: 12 months of healing), designated for micro-CT and Raman microspectroscopy analyses. A visual timeline summarizing groups, cases, analysis types, and healing time points is reported in Figure 1.

### 2.5. Optical Microscopy

The bone biopsies of cases 1–4 were fixed by immediate immersion in 10% buffered formalin and processed to obtain thin ground sections (Precise 1 Automated System; Assing, Rome, Italy). The samples were dehydrated in an ascending series of alcohol rinses and embedded in glycol-methacrylate resin (Technovit 7200 VLC; Kulzer, Wehrheim, Germany).

The specimens were sectioned using a high precision diamond disk at about 150 µm and ground down to about 30 µm with a specially designed grinding machine (Precise 1 Automated System). The obtained slices were stained with acid fuchsin and toluidine blue before the analysis.

All histologic analyses were carried out using a light microscope (Laborlux S, Leitz, Wetzlar, Germany) connected to a high-resolution video camera (3CCD, JVCKY-F55B, JVC, Yokohama, Japan) and interfaced with a monitor connected to a computer. This entire optical setup was further enhanced with a specialized digitizing pad (Matrix Vision GmbH, Oppenweiler, Germany) and histomorphometric software equipped with image capturing capabilities (Image-Pro Plus 4.5, Media Cybernetics Inc., Immagini e Computer Snc, Milano, Italy).

### 2.6. Micro-Computed Tomography

The bone biopsies of cases 5 and 6 were analyzed using X-ray micro-computed tomography (micro-CT) with a desktop device, the Bruker SkyScan 1174 μ-CT system (SkyScan-Bruker, Antwerp, Belgium). The projection parameters were set as follows: an acceleration voltage of 50 kV; a beam current of 800 µA; an aluminum filter with a thickness of 0.5 mm; a pixel size of 6.5 µm; a rotation of 180° in steps of 0.1°; and an exposure time of 6.5 s per projection. The entire scanning process lasted approximately 3 h. To reconstruct the projections into transverse slices, NRecon software (version 1.6.10.2, Bruker, Billerica, MA, USA) was employed, utilizing the following correction settings: Ring Artifact Reduction (7), Smoothing (6), and Beam Hardening correction (40%).

The 3D microstructural analysis was conducted using the BoneJ plugin within FIJI(v.1.54p) [29] to characterize the following morphological indices: the Vol%, expressed as a percentage respect to the total volume, which measures the overall mineralized volumetric density (including β-TCP/HA and newly formed bone in the apical region, as well as basal bone in the coronal region); the mean trabecular thickness (Tb.Th; μm) and the mean trabecular spacing (Tb.Sp; μm), which quantify the average thickness of trabeculae and the average distance between trabeculae, respectively. Additionally, the 3D orientation and arrangement indices—namely, the connectivity density (Conn.D; px^−3^), the degree of anisotropy (DA), and the fractal dimension (FD)—were calculated. The Conn.D parameter estimates the number of connected structures, i.e., trabeculae within a network. This connectivity measure is related to the topological Euler number (χ), with the formula: Conn.D = 1 − (χ + Δχ), where χ describes the shape or structure of a topological space, and Δχ accounts for topological changes when the structure is segmented. The input image must be three-dimensional and binary; the resulting parameter, Conn.D, represents the number of elements per unit volume, with higher values indicating better connectivity among trabeculae. The DA index quantifies the directional orientation of trabeculae, assessing whether they exhibit a preferred orientation or are randomly aligned. A DA value of 0.0 indicates complete isotropy, with no preferential directionality, while a value of 1.0 signifies a predominant orientation within the sample. The FD parameter provides a quantitative measure of the complexity of the trabecular pattern, calculated via the box-counting algorithm applied to a stack of images. This method involves overlaying grids of decreasing box sizes and counting the number of boxes containing at least one foreground voxel. As the box size diminishes, the proportion of foreground boxes increases in fractal structures. In three dimensions, FD values range from 2 (planar distribution) to 3 (fully three-dimensional distribution). The box-counting algorithm was configured with the following parameters [30]: initial box size of 48 pixels; minimum box size of 6 pixels; box scale factor of 1.2; and no grid translation.

### 2.7. Raman Microspectroscopy

After micro-CT investigation, the bone biopsies of cases 5 and 6 were also analyzed using a XploRA Nano Raman Microspectrometer (Horiba Jobin-Yvon IBH Ltd, Glasgow, Scotland) equipped with a 785-nm diode laser used as a source. All the Raman microspectroscopy (RMS) measurements were acquired by using a 5× objective (Olympus, Tokyo, Japan). The spectrometer was calibrated to the 520.7 cm^−1^ line of silicon prior to spectral acquisition. A 600 lines per mm grating was chosen. A 200 μm confocal pinhole was used for all measurements. Raman spectra were dispersed onto a 16-bit dynamic range Peltier cooled CCD detector. RMS measurements were performed on pristine granules of the employed biomaterial and on the sinus lifts, with the same parameters, in the spectral range 400–1800 cm^−1^ and accumulating 3 × 10 s at each point. As regards the sinus lifts, Raman mapping was performed on some areas of interest, including areas containing the biomaterial, areas containing the biological tissue, and interface areas. Raman maps were acquired on rectangular areas (~182 μm × 310 μm), with a step size of ~20 μm, for a total number of ~140 spectra. Representative spectra were extracted from the Raman maps, in order to compare the spectral profiles of the areas of interest. All Raman spectra were smoothed using nine smoothing points, baseline-corrected with the polynomial method (two iterations), and normalized on the 960 cm^−1^ peak (OPUS 7.5 software, Bruker Optics, Ettlingen, Germany), as a part of the pre-processing procedure. The intensity of specific Raman peaks was calculated by a local baseline-based integration procedure (OPUS 7.5 software).

### 2.8. Statistical Methods

The statistical analysis of micro-CT morphometric data and of the Raman spectra integration procedure was conducted using the Prism 10.5.0 software package (GraphPad Software, San Diego, CA, USA). All data are presented by descriptive statistics (mean, standard deviation, and range). Statistical significance among groups was assessed via one-way ANOVA followed by Tukey’s multiple comparisons test (normality and co-variance tests verified). A *p*-value of less than 0.05 was considered statistically significant.

## 3. Results

**Figure 2:**
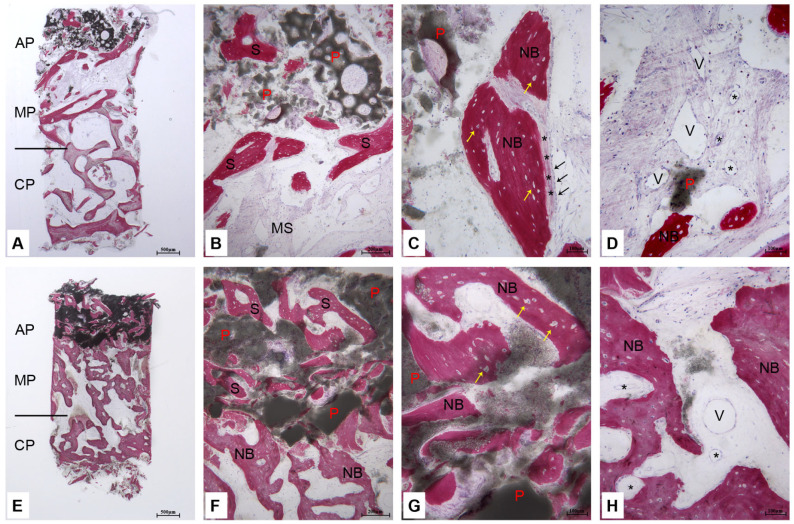
Cases 1–4: histology. (**A**–**D**) Case 1: 6 months of healing; (**A**) native bone in the crestal portion (CP), regenerated area in the middle (MP), and apical portion (AP). Toluidine blue and acid fuchsin, 12×; (**B**) apical portion: residual biomaterial particles (P) undergoing reabsorption and colonized by newly bone spicules (S) were observed in the marrow spaces (MS). Toluidine blue and acid fuchsin, 40×; (**C**) osteoblasts (black arrows) involved in the osteoid matrix deposition (*). Large osteocyte lacunae (yellow arrows) were detected in new bone trabeculae (NB). A small portion of residual biomaterial particle (P) were present near new bone (NB). Toluidine blue and acid fuchsin, 100×; (**D**) many small (*) and large blood vessels (V) were observed close to the new bone (NB) and residual biomaterial particle (P) (Toluidine blue and acid fuchsin, 100×; (E-H) case 3: 9 months of healing: (**E**) crestal portion (CP) with evidence of bone remodelling, middle portion (MP) with newly formed bone trabeculae and minimal residual biomaterial, and apical portion (AP) containing residual biomaterial and numerous newly formed bone spicules. Toluidine blue and acid fuchsin, 12×; (**F**) transition area between the middle portion, where newly formed bone trabeculae (NB) are present, and the apical portion, which contains residual biomaterial particles (P) and bone spicules (S) (Toluidine blue and acid fuchsin, 40×). (**G**) In the apical portion, residual biomaterial particles (P) are partially colonized by new bone (NB) exhibiting large osteocyte lacunae (yellow arrows). Toluidine blue and acid fuchsin, 100×; (**H**) numerous small (*) and large (V) blood vessels were observed near the newly formed bone (NB) and resorbing biomaterial (Toluidine blue and acid fuchsin, 100×).

Cases 1, 2: After 6 months of healing, the samples revealed basal bone in the crestal portion and regenerated bone area in the middle and apical portion (Figure 2A). The native bone exhibited a trabecular structure with many remodelling bone areas. Above the native bone, new bone trabeculae with a small amount of residual biomaterial were detected, suggesting it was undergoing reabsorption. In the apical portion, residual biomaterial colonized by newly bone spicules was observed (Figure 2B). Furthermore, active osteoblasts involved in new bone formation and no signs of inflammation and dense vascularization were detected (Figure 2C,D).

Cases 3, 4: After 9 months of healing, no distinct morphological features were observed between the crestal and middle portions of the sample. However, in the middle portion a small amount of biomaterial was still present among newly formed bone trabeculae. In the apical portion a considerable amount of residual biomaterial with many bone spicules was observed (Figure 2E). At a higher magnification, in the middle portion the presence of trabecular bone prevailed, whereas in the apical portion the residual biomaterial colonized by bone spicules was detected. Indeed, the new bone showed large osteocyte lacunae (Figure 2F,G). Many small and large blood vessels were observed in proximity to the newly formed bone and the resorbing biomaterial (Figure 2H).

The histomorphometric analysis of the regenerated area demonstrated that the proportion of newly formed bone was higher at 9 months compared to 6 months, while the percentage of residual particles seemed slightly higher at 6 months (statistically non-significant, see Table 2). Notably, in the native bone region (Table 3), bone percentages were higher at 9 months than at 6 months, while the proportion of marrow spaces showed a corresponding decrease.

**Figure 3:**
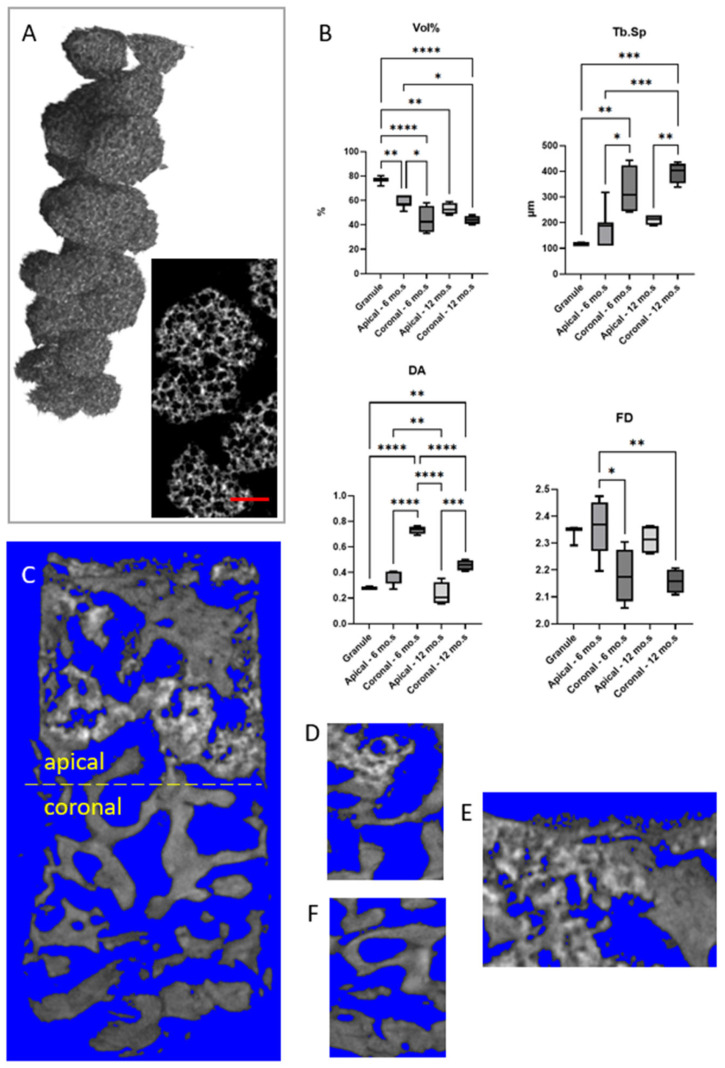
Cases 5 and 6: micro-CT imaging analysis. (**A**) 3D reconstruction and frontal slice of pristine BCP granules. Bar: 800 µm; (**B**) box-and-whisker plots illustrating the distribution of micro-CT-based morphometric parameters. Only parameters exhibiting significant differences (*p* < 0.05) between samples are reported. The upper and lower bounds of the boxes represent the 75th and 25th percentiles, respectively. The median value is indicated by a solid line. * *p* < 0.05; ** *p* < 0.01; *** *p* < 0.001; **** *p* < 0.0001. (**C**) Case 5: longitudinal section of the sample retrieved after 6 months of healing, showing the regenerated tissue in the apical region and basal bone in the coronal region; (**D**,**E**) case 5: detailed views of the apical region; (**F**) case 5: detailed view of the coronal region.

Cases 5, 6: Descriptive statistics of the morphometric parameters for both the BCP pristine granules and the Group 2 (cases 5 and 6) biopsies, analyzed via micro-CT, are summarized in Table 4. These parameters were computed using the BoneJ plugin—an extension of ImageJ (v.1.54p) specialized for bone image analysis [31]. Micro-CT images of representative BCP granules and sinus lift biopsies are presented in Figure 3A and Figure 3C–F, respectively. Box-and-whisker plots illustrating the distribution of parameters that exhibited significant differences (*p* < 0.05) between samples are shown in Figure 3B.

In accordance with the histological procedures applied to cases 1–4 from Group 1, the two samples from Group 2 were similarly virtually subdivided into two regions: the apical portion, corresponding to the regenerated area facilitated by the use of BCP granules, and the coronal portion, comprising the basal bone. Both regions in each sample were thoroughly mapped in a volumetric manner, resulting in the morphometric data presented in Table 4.

The highest values of the mineralized portion as a percentage of the total volume (Vol%—Figure 3B) were observed in the pristine BCP biomaterial (Granule), with significantly decreasing Vol% values in the regenerated (apical) regions of both biopsies (Apical (case 5–6 mo.): *p* = 0.0036; Apical (case 6–12 mo.): *p* = 0.0010). The minimum values corresponded to the basal (coronal) bone in both the biopsies collected after 6 months and that collected after 12 months from grafting (Coronal (case 5–6 mo.): *p* < 0.0001; Coronal (case 6–12 mo.): *p* < 0.0001). However, at 6 months, the regenerated portion exhibited mineralization values significantly higher than those of the basal bone (*p* = 0.0170). Conversely, in the case 6 (sample obtained after 12 months from biomaterial grafting), the mineralized content in the regenerated region showed a percentage volume that was not significantly higher than that of the corresponding basal bone (*p* > 0.05).

An opposite trend compared to the Vol% parameter was observed for the average distance between trabeculae (Tb.Sp—Figure 3B). In this case, the lowest Tb.Sp values were recorded in the pristine BCP biomaterial (granules), with increasing (though not statistically significant—*p* > 0.05) values in the regenerated (apical) regions of both biopsies. The highest Tb.Sp values corresponded to the basal (coronal) bone in both biopsies collected after 6 months and after 12 months from grafting (Coronal (case 5–6 months): *p* = 0.0029; Coronal (case 6–12 months): *p* = 0.0001). Consistently, at both 6 months (*p* = 0.0155) and 12 months (*p* = 0.0041), the regenerated regions exhibited significantly lower Tb.Sp values compared to the basal bone.

The degree of anisotropy (DA—Figure 3B) also followed a trend similar to that observed for the Tb.Sp parameter, with an increase (though not statistically significant—*p* > 0.05) in the DA from the granules to the apical regenerated regions. The highest DA values were observed in the basal (coronal) bone in both biopsies collected after 6 months and after 12 months from grafting (Coronal (case 5–6 months): *p* < 0.0001; Coronal (case 6–12 months): *p* = 0.0045). Consistently, at both 6 months (*p* < 0.0001) and 12 months (*p* = 0.0001), the regenerated regions showed significantly lower DA values compared to the basal bone.

Finally, a different pattern emerges regarding the analysis of Fractal Dimension (FD—Figure 3B). The BCP biomaterial did not exhibit significant differences (*p* > 0.05) compared to the values observed in both the apical and coronal regions of both biopsies. However, the regenerated area in the apical region at 6 months appears to have a significantly higher fractal dimension than the corresponding basal bone in both biopsies (Apical (case 5–6 months) vs. Coronal (case 5–6 months): *p* = 0.0152; Apical (case 5–6 months) vs. Coronal (case 6–12 months): *p* = 0.0066).

**Figure 4:**
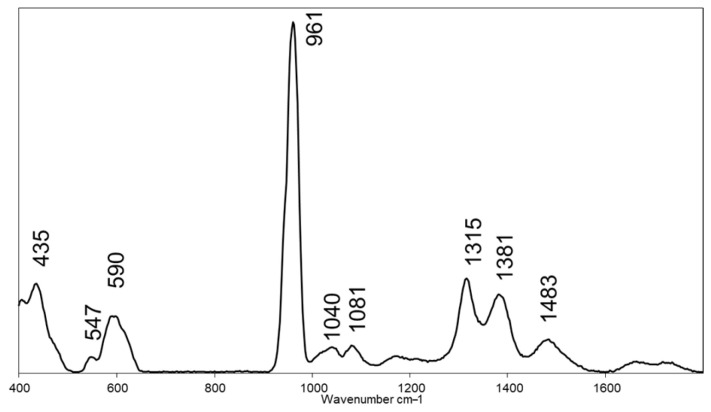
Raman microspectroscopy: spectral profile of pristine granules of biomaterial, composed of 70% β-tricalcium phosphate (β-TCP) and 30% hydroxyapatite (HA). The Raman spectrum is displayed in the 400–1800 cm^−1^ range; the position of the main peaks of interest is labelled (wavenumbers, cm^−1^).

The Raman spectrum acquired on granules of the pristine biomaterial, composed of 70% β-tricalcium phosphate (β-TCP) and 30% hydroxyapatite (HA), was characterized mainly by the internal modes of the PO_4_^3−^ anion (Figure 4) [32]. In fact, the following peaks were identified: 435 cm^−1^ (ν_2_ of the PO_4_^3−^ groups of HA and β-TCP) [33]; 547 cm^−1^ (β phase of TCP) [34]; 590 cm^−1^ (ν_4_ of the PO_4_^3−^ groups of HA and β-TCP); 961 cm^−1^ (ν_1_ of the PO_4_^3−^ groups of HA [33]; in β-TCP it is shifted towards higher Raman shifts (~970 cm^−1^), with a shoulder at ~949 cm^−1^ [35]; 1040 cm^−1^ and 1080 cm^−1^ (ν_3_ of the PO_4_^3−^ groups, respectively, of HA and β-TCP) [36]; 1315 cm^−1^, 1381 cm^−1^, and 1480 cm^−1^ (unassigned, but present in β-TCP in other papers) [37].

**Figure 5:**
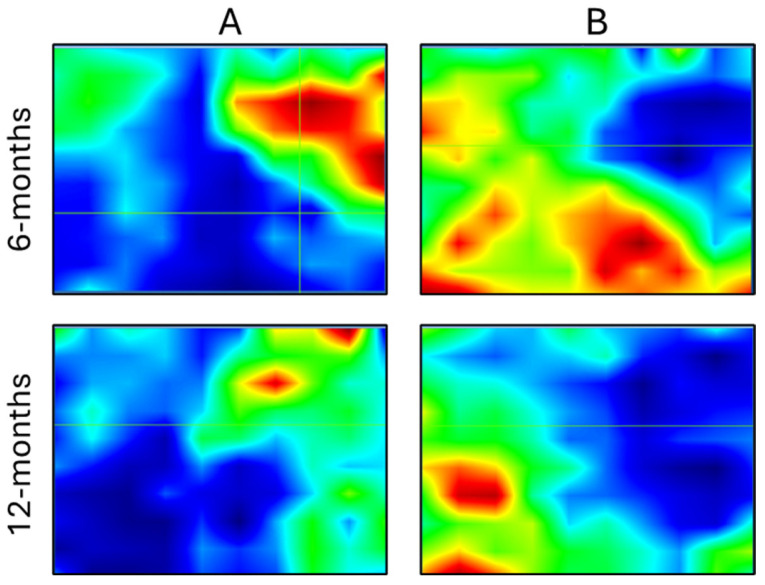
Cases 5 and 6: Raman microspectroscopy. Raman maps acquired on the interface between the biomaterial and the newly formed tissue showing the distribution of the bands at: (**A**) 960 cm^−1^ (assigned to HA) and (**B**) 1660 cm^−1^ (assigned to the biological tissue), after 6 (case 5) and 12 (case 6) months of healing. Black/blue color corresponds to the lowest values, green intermediate, and red/dark red to the highest values.

Based on this evidence, a typical Raman mapping analysis was performed on sinus lifts, at the interface between the biomaterial and the newly formed tissue, in cases 5 and 6, i.e., after 6 and 12 months, respectively. To make this, Raman maps were integrated under the peaks centered at 960 cm^−1^ (assigned to HA in the biomaterial) and 1660 cm^−1^ (assigned to the Amide I band of proteins and hence attributable to the biological tissue) (Figure 5). The analysis clearly evidenced a complementary distribution of the inorganic (right part of the Raman maps) and organic (left part of the Raman maps) components. Interestingly, at the 12-month time point, the area occupied by HA is greater, but with a lower intensity: this evidence let hypothesize and absorption of the biomaterial and the contemporary formation of newly bone tissue.

**Figure 6:**
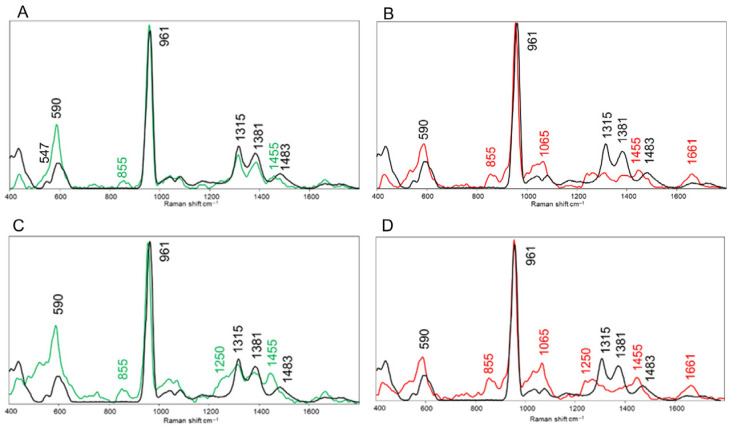
Cases 5 and 6: Raman microspectroscopy. Representative Raman spectrum of the pristine biomaterial (black line) superimposed with (**A**) (case 5) the representative Raman spectrum of the biomaterial within the sinus lift sample after 6 months (green line), (**B**) (case 5) the representative Raman spectrum of the newly formed tissue within the sinus lift sample after 6 months (red line), (**C**) (case 6) the representative Raman spectrum of the biomaterial within the sinus lift sample after 12 months (green line), and (**D**) (case 6) the representative Raman spectrum of the newly formed tissue within the sinus lift sample after 12 months (red line). Raman spectra are displayed in the 400–1800 cm^−1^ range; the position of the main peaks of interest is labelled (wavenumbers, cm^−1^).

To better evaluate the absorption of the biomaterial after 6 and 12 months, representative Raman spectra extracted from the areas containing the biomaterial and the newly formed tissue were analyzed. Figure 6A,B refers to case 5, corresponding to the 6-month time point. Figure 6A displays the comparison between the spectrum of the pristine biomaterial (black line) and the one representative of an area containing biomaterial (green line). As regards the sinus lift sample, (i) the peak centered at 547 cm^−1^, assigned to the β phase of TCP [34], is absent; (ii) the intensity of the peak centered at 590 cm^−1^, assigned to ν_4_ of the PO_4_^3−^ groups of HA and β-TCP [33], is higher; (iii) the typical peaks of pristine β-TCP, 1315 cm^−1^, 1380 cm^−1^, and 1483 cm^−1^, are still visible, and (iv) a small peak centered at 1455 cm^−1^, assigned to CH_2_ bending modes, is raising. Similar differences are evidenced in Figure 6B, by comparing the spectrum of the pristine biomaterial (black line), with the one representative of an area containing newly formed biological tissue (red line). In addition, in the spectrum of the sinus lift sample, (i) the peaks centered at 855 cm^−1^, assigned to ring vibrations of proline [38], and 1065 cm^−1^, assigned to the stretching of CO_3_^2−^ groups [39], appeared, partly covering the peaks assigned to ν_3_ of the PO_4_^3−^ groups of HA and β-TCP; (ii) two small peaks are centered at 1455 cm^−1^, assigned to the bending of CH_2_ groups [40], and 1661 cm^−1^, due to the Amide I band raise [40], and (iii) the typical peaks of pristine β-TCP, 1315 cm^−1^, 1380 cm^−1^, and 1483 cm^−1^, have almost completely disappeared.

The same analysis was performed on case 6, i.e., the 12-month sinus lift sample (Figure 6C,D). All the differences highlighted in the 6-month sample were visible also at 12 months, at a higher degree. In fact, also in the biomaterial area of the sinus lift (Figure 6C), it was possible to detect the peaks centered at 855 cm^−1^ and 1455 cm^−1^, previously detected only in the area of the biological tissue; moreover, in both the analyzed regions, another peak was visible respect to the spectrum of the pristine biomaterial: the peak centered at 1250 cm^−1^ and assigned to Amide III [40]; finally, in the representative spectrum of the newly formed biological tissue (Figure 6D), the three typical peaks of pristine β-TCP, 1315 cm^−1^, 1380 cm^−1^, and 1483 cm^−1^, completely disappeared.

**Figure 7:**
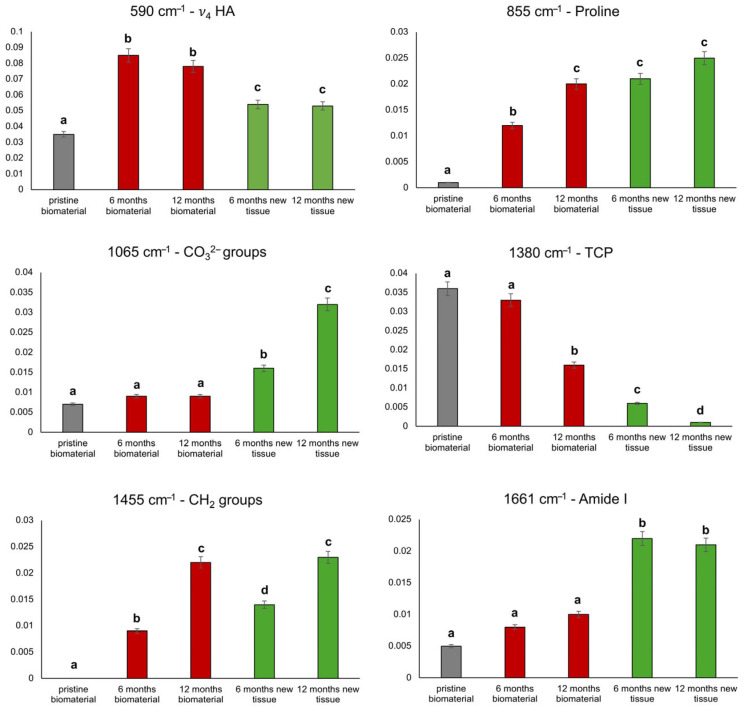
Cases 5 and 6: histograms show the numerical variation of the intensities of the 590, 855, 1065, 1380, 1455, and 1661 cm^−1^ peaks, respective to the pristine biomaterial. Data are represented as mean ± SD. Different letters over histograms indicate statistically significant difference among groups (one-way ANOVA and Tukey’s multiple comparison test). Statistical significance was set at *p* < 0.05.

In order to provide a semi-quantitative analysis of the described results, some relevant Raman peaks were integrated and their intensity was calculated and compared among the five experimental groups. The results are shown in Figure 7.

## 4. Discussion

In this case series, we analyzed biopsies obtained from maxillary sinus augmentation procedures performed using the classical lateral window technique, employing porous biphasic calcium phosphate (BCP) granules composed of 70% beta-tricalcium phosphate (β-TCP) and 30% hydroxyapatite (HA). All biopsies were collected by a single surgeon and examined through a multidisciplinary approach including histological analysis, micro-CT, and Raman microspectroscopy to evaluate the evolution of regenerated bone tissue over time.

Regarding the cases, six human biopsies from sinus lift augmentations were analyzed, divided into three healing time groups of 6, 9, and 12 months. Sample preparation involved preservation in formalin, followed by sectioning and staining for histological analysis (Group 1—cases 1–4), and micro-CT and Raman microspectroscopy on the remaining samples (Group 2—cases 5 and 6).

Histological analysis demonstrated a more distinct differentiation between native and regenerated bone regions at 6 months of healing compared to nine months. The coronal portion predominantly contained native trabecular bone characterized by ongoing remodeling activity, while the apical region showed evidence of new bone formation, with newly formed trabeculae extending into the area. The presence of residual biomaterial was present but not significant, also indicating the presence of active resorption processes. Notably, in the apical zone, residual biomaterial was colonized by new bone spicules, suggesting successful integration and osteoconductivity of the BCP granules. Additionally, dense vascularization, active osteoblasts, and the absence of inflammatory signs collectively support the biocompatibility and regenerative potential of the material used. The histomorphometric analysis confirmed these observations, showing higher percentages of new bone in the regenerated area after 9 months compared to 6 months of healing. These findings underscore the material’s capacity to facilitate bone regeneration while being partially resorbed and replaced by new, functional bone tissue [41].

Micro-CT analysis provided 3D morphometric data, including the percentage of mineralized volume, average trabecular thickness, trabecular separation, and parameters of structural complexity such as connectivity density, anisotropy index, and fractal dimension. These parameters were assessed not only in the two sinus lift augmented biopsies of cases 5 and 6 but also in the pristine BCP granules.

A key feature of the pristine BCP granules used as scaffolds is their three-dimensional interconnected porous structure, exhibiting an actual porosity of 20–30% and an average pore size of approximately 120 µm. This interconnected porosity was designed to facilitate cellular colonization and vascularization in vivo [42]. The mesh-like architecture aimed to promote cell penetration into the graft, while micropores were intended to support capillary infiltration even in the innermost regions, thereby delivering essential growth factors for new bone formation and enhancing the osteoinductive capacity of the graft.

Micro-CT results from case 5 (biopsy taken 6 months post-grafting) showed that the regenerated (apical) portion exhibited a significantly higher mineralized content compared to the native (coronal) tissue, with increased trabecular density and connectivity. Over time, a partial but progressive resorption of the biomaterial and maturation of the new bone tissue were observed, as evidenced by case 6 (12-month post-grafting biopsy). Overall, the parameters measured in the apical (engineered) regions—namely, the percentage volume of mineralized tissue, pore size, and degree of anisotropy of the trabecular structures—are consistent with values reported in the literature for comparable grafting durations following the application of other biomaterials (see Table S1 in the Supplementary Materials).

The micro-CT findings were corroborated by Raman microspectroscopy, performed on the same cases 5 and 6, which provided molecular insights into the chemical composition of both the biomaterial and the regenerated tissue. Raman analysis enabled discrimination between β-TCP and HA and monitored the resorption of BCP and the progression of bone formation over time. The spectra highlighted a gradual disappearance of characteristic β-TCP peaks and the emergence of molecular markers typical of mature bone tissue.

## 5. Conclusions

In conclusion, this case series collectively suggests that BCP granules facilitate osteoconduction and undergo gradual resorption, with regenerative processes extending well beyond 6 months post-grafting and remaining active up to 12 months after biomaterial placement. Once positioned within the defect site, BCP granules rapidly dissolve owing to their β-TCP component, which releases calcium ions (Ca^2+^), thereby supporting ongoing bone regeneration. These preliminary findings are subject to certain limitations concerning statistical significance and highlight the necessity for further validation through larger-scale follow-up studies with more extensive sample sizes. Such studies are essential to confirm the observed temporal dynamics and to enhance the understanding of the long-term regenerative potential of BCP granules.

## Figures and Tables

**Table 1:** Demographic data of the study and patient medical records.

Case	Gender	Age	Healing Time	Patients’ Medical Records
Case 1	F	45	6 months	The patient had undergone an extraction of a tooth 1.6 approximately 1 year earlier due to a root fracture. The residual basal bone was insufficient for implant placement, necessitating maxillary sinus floor elevation.
Case 2	M	65	6 months	The patient presented with edentulism of the upper jaw, requiring implant rehabilitation through bilateral maxillary sinus floor elevation (bone harvested from the left side).
Case 3	F	53	9 months	Tooth 1.4 had been missing for approximately 7 years, also requiring sinus floor elevation due to insufficient residual bone volume.
Case 4	F	60	9 months	There was edentulism in the molar region of the right maxilla, along with carious lesions affecting the premolars, which were subsequently extracted.
Case 5	M	65	6 months	The patient presented with edentulism of the upper jaw, requiring implant rehabilitation through bilateral maxillary sinus floor elevation (bone harvested from the right side).
Case 6	M	62	12 months	The right posterior maxillary region had been edentulous for approximately 5 years. To enable implant placement and restore function, sinus floor elevation was required.

**Table 2:** Histomorphometric analysis of the regenerated area to quantify the relative proportions of newly formed bone, residual graft particles, and marrow spaces. Data are reported for each individual case and as mean ± standard deviation (SD), expressed as percentages according to healing time.

	New Bone (%)	Residual Particles (%)	Marrow Spaces (%)
Case 1–6 months	12.54	10.18	77.29
Case 2–6 months	18.27	29.88	51.85
Mean ± SD	15.40 ± 4.05	20.03 ± 13.93	64.56 ± 17.99
Case 3–9 months	28.06	14.62	57.32
Case 4–9 months	25.43	23.29	51.29
Mean ± SD	26.74 ± 1.86	18.95 ± 6.13	54.30 ± 4.27

**Table 3:** Histomorphometric analysis of the native bone region, quantifying the proportions of bone and marrow spaces. Data are presented for each individual case, as well as mean ± standard deviation (SD), expressed as percentages according to healing time.

	Bone (%)	Marrow Spaces (%)
Case 1–6 months	37.09	62.91
Case 2–6 months	31.10	68.89
Mean ± SD	34.09 ± 4.22	65.90 ± 4.22
Case 3–9 months	40.74	59.26
Case 4–9 months	50.52	49.48
Mean ± SD	45.63 ± 6.91	54.37 ± 6.91

**Table 4:** Descriptive statistics of the micro-CT morphometric parameters: while the coronal region contains only basal bone, the apical region exhibits regenerated bone in conjunction with residual BCP biomaterial. Mean ± standard deviation (range: minimum ÷ maximum).

Morphometric Parameter	Granule	Case 5 Apical (6 mo.)	Case 5 Coronal (6 mo.)	Case 6 Apical (12 mo.)	Case 6 Coronal (12 mo.)
Vol% [%]	76 ± 4 (72 ÷ 80)	58 ± 5 (51 ÷ 64)	44 ± 11 (33 ÷ 58)	53 ± 5 (48 ÷ 59)	44 ± 3 (40 ÷ 48)
Tb.Th [µm]	195 ± 13 (182 ÷ 208)	211 ± 30 (182 ÷ 254)	265 ± 81 (202 ÷ 371)	218 ± 28 (182 ÷ 241)	284 ± 37 (254 ÷ 338)
Tb.Sp [µm]	119 ± 4 (117 ÷ 124)	187 ± 70 (111 ÷ 319)	325 ± 96 (241 ÷ 442)	211 ± 20 (189 ÷ 228)	395 ± 41 (338 ÷ 436)
Conn.D (×10^−5^) [px^−3^]	0.75 ± 0.65 (0.12 ÷ 1.42)	1.50 ± 1.02 (0.55 ÷ 3.11)	0.51 ± 0.20 (0.36 ÷ 0.79)	1.14 ± 0.37 (0.77 ÷ 1.70)	0.39 ± 0.19 (0.18 ÷ 0.63)
DA	0.28 ± 0.01 (0.28 ÷ 0.29)	0.37 ± 0.05 (0.27 ÷ 0.41)	0.73 ± 0.03 (0.69 ÷ 0.77)	0.23 ± 0.09 (0.16 ÷ 0.36)	0.46 ± 0.04 (0.41 ÷ 0.50)
FD	2.33 ± 0.04 (2.29 ÷ 2.36)	2.36 ± 0.10 (2.20 ÷ 2.47)	2.18 ± 0.10 (2.06 ÷ 2.30)	2.31 ± 0.06 (2.26 ÷ 2.36)	2.16 ± 0.05 (2.11 ÷ 2.21)

## Data Availability

All the original contributions presented in this study are included in the article. Further inquiries can be directed to the corresponding author.

## Supplementary Materials

The following supporting information can be downloaded at: https://www.mdpi.com/article/10.3390/bioengineering12080848/s1, Table S1: Biomaterials performance in sinus lift augmentation—microarchitecture in clinical cases.

## Abbreviations

The following abbreviations are used in this manuscript:

β-TCP	Beta tricalcium phosphate
Micro-CT	Micro-computed tomography
BCP	Biphasic calcium phosphate
RMS	Raman microspectroscopy
Vol%	Ratio between mineralized volume and total volume
Tb.Th	Mean trabecular thickness
Tb.Sp	Mean trabecular spacing
Conn.D	Connectivity density
DA	Anisotropy degree
FD	Fractal dimension
CP	Crestal portion
MP	Middle portion
AP	Apical portion
