# Supplementary material for: Healing Kinetics of Sinus Lift Augmentation Using Biphasic Calcium Phosphate Granules: A Case Series in Humans
Source: Bioengineering (Basel). 2025 Aug 6;12(8):848. doi: 10.3390/bioengineering12080848 (PMC12383563; doi:10.3390/bioengineering12080848)
Supplement: Supplementary file 1 [file bioengineering-12-00848-s001.zip › bioengineering-3733368-supplementary.pdf]

Healing Kinetics of Sinus Lift Augmentation Using Biphasic Calcium Phosphate Granules: A Case Series in Humans

Michele Furlani, Valentina Notarstefano, Nicole Riberti, Emira D'Amico, Tania Vanessa Pierfelice, Carlo Mangano, Elisabetta Giorgini, Giovanna Iezzi and Alessandra Giuliani

Supplementary Material

Table S1. Biomaterials performance in Sinus Lift Augmentation- Microarchitecture in Clinical cases.

Biomaterial	NB (vol.%)	ResBio (vol.%)	TB.Th (µm)	TB.Sp (µm)	Conn.D(mm-1)	FD	DA	t (mo.s)	Reference
Bio-Oss ®	37.2 (4.7)	36.6 (3.4)	-	-	-	-	-	6	DOI: 10.1111/cid.13331
Bio-Oss ® + Bio-Gide® collagen membrane	36.6 (3.4)	27.2 (5)	-	-	-	-	-	6	DOI: 10.1111/cid.13331
Bio-Oss ®	34.11		50	60	-	-	-	6	DOI: 10.3390/medicina60111834
Cerabone®	66.06%		80	40	-	-	-	6	DOI: 10.3390/medicina60111834
human bone allograft (MHBA)	37.2	19.55	-	-	-	-	-	5–13	DOI: 10.1111/clr.12225
platelet-rich fibrin (A-PRF) + serum albumin-coated bone allograft (SACBA)	21.2	0	184	520	1988	-	-	6	DOI: 10.3390/ma14071810
Autologous + Bio-Oss ®	48.2 (21.6–90.0)		234 (159–556)	186 (97–281)	-	-	0.139 (0.119–0.206)	6–8	DOI: 10.1111/clr.12380
Autologous + BoneCeramic®	43.2 (13.5–67.4)		185 (110–220)	195 (100–330)	-	-	0.127 (0.112–0.149)	6–8	DOI: 10.1111/clr.12380
Cortico-cancellous porcine bone (mp3®)	62.0(6.2)		105 (14)	64 (9)	-	-	-	6	DOI 10.1007/s00784-017-2139-6
Biphasic Calcium Phosphate (HA/b-TCP 30/70) - Blocks	18.9 (6.3)	27.3 (7.5)	50.3 (4.8)	-	-	-	0.617 (0.090)	5-6	DOI: 10.1097/ID.0000000000000363
Biphasic Calcium Phosphate Scaffolds (HA/b-TCP 30/70) - Granules	26.5 (8.9)	19.7 (3.6)	55.0 (3.5)	-	-	-	0.495 (0.158)	5-6	DOI: 10.1097/ID.0000000000000363
Pure Biocoral	30.5	1.9	69	144	-	-	0.19	6-7	DOI 10.1111/cid.12039
Biocoral + TCP	16.8	35.2	189	148	-	-	0.16	6-7	DOI 10.1111/cid.12039
Biphasic Calcium Phosphate (HA/b-TCP 30/70) - Blocks	26.0	2.9	55	137	-	-	0.13	6-7	DOI 10.1111/cid.12039

NB (vol.%): newly formed bone (volume percentage); ResBio (vol.%): residual biomaterial (volume percentage); TB.Th (µm): mean trabecular thickness; TB.Sp (µm): mean trabecular spacing; Conn.D (mm^{-1}): connectivity density; FD: fractal dimension; DA: anisotropy degree; t (mo.s): healing time (months).
